# Withaferin A Inhibits Neutrophil Adhesion, Migration, and Respiratory Burst and Promotes Timely Neutrophil Apoptosis

**DOI:** 10.3389/fvets.2022.900453

**Published:** 2022-06-17

**Authors:** Rosemary L. Bayless, M. Katie Sheats, Samuel L. Jones

**Affiliations:** ^1^Department of Clinical Sciences, College of Veterinary Medicine, North Carolina State University, Raleigh, NC, United States; ^2^Comparative Medicine Institute, North Carolina State University, Raleigh, NC, United States

**Keywords:** neutrophil (PMN), Withaferin A (PubChem CID: 265237), equine, inflammation, phytochemical, veterinary medicine, novel therapeutic

## Abstract

Neutrophils play a major role in many equine conditions, including equine asthma, laminitis, and intestinal ischemia and reperfusion injury, and therefore represent an attractive target for innovative therapeutic approaches. Novel strategies for reducing neutrophilic inflammation include modulation of neutrophil functions and lifespan. Withaferin A (WFA) is a phytochemical with well-established *in vitro* and *in vivo* anti-inflammatory properties, but its direct effects on neutrophils are largely unknown. We hypothesized that WFA would inhibit adhesion, migration, and respiratory burst by equine neutrophils and promote timely apoptosis of primed equine neutrophils. Consistent with this hypothesis, our data show that WFA causes a significant, concentration-dependent inhibition of equine neutrophil adhesion, migration, and respiratory burst in response to diverse stimuli. Further, WFA treatment increased apoptosis of equine neutrophils exposed to GM-CSF for 24 h. This pro-apoptotic effect of WFA was not observed in unprimed neutrophils, nor at the 2-h time point relevant to our functional neutrophil experiments. Our data demonstrate that WFA may reduce neutrophil-mediated inflammation through multiple mechanisms, including suppression of inflammatory responses and promotion of apoptosis. Additional research is needed to elucidate the molecular mechanisms for these effects and evaluate the potential clinical use of WFA in veterinary and human patients.

## Introduction

While neutrophils are an integral part of the innate immune system, dysregulation of neutrophil functions can cause substantial damage to host tissue. In horses, neutrophilic inflammation has been implicated in the pathophysiology of diseases that can be acutely life-threatening or compromise quality of life and/or athletic performance (1–6). The common role of neutrophils in these conditions makes these cells a potential target for treating inflammatory conditions. There is also ample evidence that neutrophils contribute to disease in humans and other veterinary species, expanding the potential impact of neutrophil-targeted therapeutic strategies (7–11).

Neutrophil participation in immune responses can be characterized by a series of interrelated and overlapping events; these processes, from initial neutrophil recruitment to microbicidal activity at the site of inflammation, represent potential opportunities for collateral tissue injury. Adhesion of circulating neutrophils to endothelium is an early requisite step for neutrophil trafficking to sites of inflammation and for preparing neutrophils to respond to subsequent inflammatory mediators (12, 13). After adhesion, neutrophils extravasate across the endothelium, follow chemotactic gradients through interstitium, and, in some cases, undergo transepithelial migration (14). The movement of neutrophils across epithelium can compromise intestinal barrier function (15–17) and increase pulmonary epithelial permeability (18). Upon reaching target tissues, neutrophils perform effector functions, such as respiratory burst, degranulation, phagocytosis, and extrusion of neutrophil extracellular traps (NETs) (19). Though these effector functions are designed to benefit host defense against pathogens or healing from injury, inappropriate or prolonged release of reactive oxygen species, granule cytotoxic proteins, and NETs also cause damage of host tissue, exacerbating disease pathology (19). Because adhesion, migration, and respiratory burst are key steps in neutrophilic inflammation, they also represent promising therapeutic targets to reduce neutrophil-mediated tissue injury (19).

Under healthy conditions, the detrimental potential of neutrophils is controlled by constitutive apoptosis (20), limiting their average lifespan in circulation to 25 h (21). However, once exposed to inflammatory molecules, such as granulocyte-macrophage colony-stimulating factor (GM-CSF) and lipopolysaccharide (LPS), neutrophils become primed, the functional lifespan of neutrophils is extended (22, 23), and neutrophil apoptosis becomes a critical step in the resolution of inflammation. Apoptotic neutrophils have decreased expression of proinflammatory genes (24) and reduced capacity to perform deleterious effect or functions (25). Additionally, phagocytosis of apoptotic neutrophils by macrophages induces a shift toward an anti-inflammatory macrophage phenotype which promotes resolution of inflammation (26). Importantly, delayed neutrophil apoptosis has been documented in a variety of inflammatory conditions affecting horses and humans, and may contribute to exacerbation of disease pathophysiology (27–34). Accordingly, therapies that promote timely neutrophil apoptosis could hasten resolution of inflammation and recovery/remission from clinical disease.

Regulation of neutrophil effector functions and apoptosis are potential strategies for novel anti-inflammatory therapies (35–40). However, there are currently no therapies approved by the US Food and Drug Administration for these indications, underscoring the importance of further research to identify and characterize potential novel neutrophil-directed anti-inflammatory treatments. A growing number of these experimental anti-inflammatory agents are natural products, often isolated from plants used in traditional medicine (37–40).

Withaferin A (WFA) is a steroidal lactone derived from the *Withania somnifera* plant, which has been used for millennia in Ayurvedic medicine to treat many different conditions (41). More recently, WFA has been recognized as a primary bioactive constituent of this plant, and *in vitro* and *in vivo* studies have documented beneficial effects of WFA for inflammatory diseases and multiple types of cancer (42–50). Neutrophils are central to the pathophysiology of several of these diseases, including acute lung injury, acute pancreatitis, and gout (51–56). In animal models of disease, treatment with WFA was associated with both clinical improvement and reduced neutrophil infiltration into relevant tissues (42–44, 57). These *in vivo* findings are further supported by an *in vitro* study showing that WFA reduced neutrophil transmigration across an endothelial monolayer toward *Escherichia coli* (58). However, a thorough investigation of specific effects of WFA on neutrophil functions, including adhesion, migration, and respiratory burst in response to diverse stimuli, has not been published.

In addition to being anti-inflammatory, WFA is also reported to induce apoptosis in a variety of cancer cell lines (59–66). Interestingly, WFA has no effect on apoptosis of normal lymphocytes and monocytes (67, 68) and is actually anti-apoptotic in different types of non-neoplastic cells, including cardiomyocytes (69, 70), pancreatic islet cells (71), renal epithelium (57, 72), and cells within the spinal cord (73). Although the effect of WFA on neutrophil apoptosis has not been described, promotion of neutrophil apoptosis by WFA could help to explain previous reports of the benefits of WFA for treating inflammatory conditions.

Based on previous evidence of WFA's anti-inflammatory and pro-apoptotic properties, we hypothesized that WFA would inhibit neutrophil adhesion, migration, and respiratory burst and would promote apoptosis of primed neutrophils. Here we report that WFA significantly attenuated neutrophil functions without affecting short-term neutrophil viability. Additionally, WFA treatment promoted timely apoptosis in GM-CSF-primed neutrophils. These findings provide strong *in vitro* support for WFA as a therapeutic for neutrophil-mediated diseases, which could have important implications for both veterinary and human patients.

## Materials and Methods

### Reagents

Withaferin A (WFA), leukotriene B4 (LTB4), and platelet activating factor (PAF; C-16) were from Cayman Chemical (Ann Arbor, MI, USA); dimethyl sulfoxide (DMSO), ethanol (EtOH), recombinant human interleukin-8 (IL-8), phorbol 12-myristate 13-acetate (PMA), bovine serum albumin (BSA), rabbit anti-bovine albumin antiserum (anti-BSA), lipopolysaccharide (LPS) from *Escherichia coli* (O111:B4), luminol sodium salt, and penicillin-streptomycin were from MilliporeSigma (Burlington, MA, USA); recombinant equine granulocyte-macrophage colony-stimulating factor (GM-CSF) was from Kingfisher Biotech, Inc. (Saint Paul, MN, USA); heat-inactivated fetal bovine serum (FBS) and Roswell Park Memorial Institute (RPMI) 1640 Medium were from Thermo Fisher Scientific (Waltham, MA, USA); Hank's Balanced Salt Solution (HBSS) was from Fisher Scientific (Hampton, NH, USA).

Crystalline WFA was dissolved in anhydrous DMSO, producing a 35.4 mM WFA stock solution that was aliquoted and stored at −20°C. WFA treatment solutions were prepared fresh each experiment by diluting WFA stock in the assay media. The treatment vehicle control (VC) was equivalent to the concentration of DMSO in the highest concentration of WFA for each assay.

### Horses

University-owned horses at North Carolina State University were blood donors for this study. All horses were considered healthy based on physical examination and history. The blood collection protocol was approved by the North Carolina State University Institutional Animal Care and Use Committee (IACUC protocol #19-779).

### Neutrophil Isolation

Neutrophils were isolated from equine whole blood as previously described (74, 75). Briefly, heparinized whole blood was collected via jugular venipuncture. Whole blood was placed into sterile conical tubes for 1 h at room temperature to allow erythrocyte sedimentation. Leukocyte-rich plasma was layered on top of Ficoll-Paque Plus (GE Healthcare, Sweden) in a 2:1 ratio and centrifuged at 610 × g for 20 min. Residual erythrocytes were removed from the neutrophil pellet via hypotonic lysis, and the neutrophil concentration, viability (≥98%), and purity (≥95%) were established using a hemocytometer and trypan blue stain. Neutrophils were resuspended in assay media as described.

### Adhesion

Isolated neutrophils were resuspended in HBSS and loaded with calcein-AM (2 μg/mL; Fisher Scientific) for 30 min at room temperature prior to resuspension in HBSS containing 1 mM CaCl_2_, 1 mM MgCl_2_, and 5% FBS (HBSS^++^ + 5% FBS). Neutrophils (1 x 10^5^) and treatment solutions (WFA, VC, or media) were combined in wells of high binding plates coated with FBS and allowed to settle for 10 min at 37°C. Neutrophils were stimulated with interleukin-8 (IL-8; 100 ng/mL), leukotriene B4 (LTB4; 100 nM), platelet activating factor (PAF; 100 nM), phorbol 12-myristate 13-acetate (PMA; 10 ng/mL), or respective stimulus controls (IL-8 & PMA: HBSS; LTB4 & PAF: 0.03% EtOH) and incubated for 3 min (IL-8, LTB4, PAF) or 30 min (PMA) at 37°C. For insoluble immune complex (IIC) stimulation, BSA-coated plates were incubated with rabbit anti-BSA antibody for 2 h at 37°C to reach IIC densities of 5 μg and 20 μg per well. Unstimulated wells were coated only with 100 μg/mL BSA. The plates were washed to remove non-immobilized IIC, and neutrophils (1 x 10^5^) and treatment solutions were combined in wells and incubated for 30 min at 37°C. All treatment/stimulation conditions were performed in triplicate. Fluorescence was measured at the end of the incubation period using a plate reader (FilterMax F5, Molecular Devices) and in between serial washing of the wells with HBSS. Percent adhesion was calculated by dividing fluorescence after washing by the initial fluorescence, and the wash at which adhesion of media control, unstimulated neutrophils was approximately 10% was used for data analysis.

### Migration

Neutrophils were loaded with calcein-AM and resuspended in HBSS^++^ + 2% FBS as described for adhesion assays and pre-treated with WFA, VC, or media for 30 min at 37°C. Chemoattractants (IL-8 [100 ng/mL], LTB4 [10 nM], or PAF [100 nM]), chemoattractant controls (HBSS, 0.003% EtOH, or 0.03% EtOH, respectively), or media were added to the lower wells of a ChemoTx system (Neuro Probe) and overlaid by a membrane with 5 μm pores. Pre-treated neutrophils (1 x 10^4^ for IL-8, 4 x 10^4^ for LTB4 or PAF) were loaded on top of the membrane. All treatment/stimulation conditions were performed in triplicate. Control (100% migration) wells consisted of the same number of neutrophils placed directly into the lower wells, representative of the fluorescence if all the neutrophils had migrated. After incubation for 1 h at 37°C, non-migrated cells remaining above the membrane were removed using a scraper. EDTA (0.5 M; Thermo Fisher Scientific) was added to membranes above each well and incubated for 10 min at room temperature before centrifugation of the plate for 5 min at 100 × g. The membrane was removed, and fluorescence of each microplate well was determined using a plate reader. Percent migration was calculated by dividing fluorescence of experiment wells by the mean fluorescence of 100% migration control wells.

### Respiratory Burst

Neutrophils were resuspended in HBSS^++^ + 5% FBS and pre-treated with WFA, VC, or media for 30 min at 37°C. Where indicated, priming with GM-CSF (1 ng/mL) was performed during the pre-treatment period. Pre-treated neutrophils (1 x 10^5^ for PMA, 3 x 10^5^ for GM-CSF/LPS) were plated in FBS-coated wells, and luminol sodium salt (1 mM) was added to each well. Once stimuli (100 ng/mL LPS or 100 ng/mL PMA) or the appropriate stimulus control (HBSS or 0.0006% DMSO, respectively) had been added to each well, the plate was incubated at 37°C in a plate reader, and luminescence values were recorded every 5 min (t = 0–90 min). In separate experiments, pre-treated neutrophils (3 x 10^5^) were added to wells coated with 5 μg insoluble immune complexes (IIC composed of BSA/anti-BSA antibodies; wells coated with 100 μg/mL BSA alone served as unstimulated conditions). These plates were also incubated at 37°C with luminescence values recorded every 5 min for 90 min. The timepoint at which maximum luminescence of media control, stimulated neutrophils was determined and used to establish the timepoint for data analysis for each stimuli: GM-CSF/LPS: 40 min, PMA: 60 min, IIC: 30 min (Supplementary Figure S1) (74). Luminescence values for WFA-treated neutrophils were expressed relative to the luminescence value of neutrophils treated with VC, yielding percent inhibition of respiratory burst relative to treatment VC. The effect of WFA on the overall respiratory burst was also evaluated by calculating the area under the curve (AUC) during the 90-min luminescence monitoring period.

### Detection of Neutrophil Apoptosis and Viability

Isolated neutrophils resuspended (1 x 10^6^/mL) in RPMI with 10% FBS, 100 U/mL penicillin, and 100 μg/mL streptomycin were simultaneously treated with WFA, VC, or media and primed with GM-CSF (50 ng/mL) or left unprimed. Primed and unprimed neutrophils were also treated with staurosporine (1 μM) as a positive control for induction of neutrophil apoptosis (76). After 2- or 24-hours incubation at 37°C/5% CO_2_, neutrophils were washed with cold HBSS and stained with Alexa Fluor® 488 annexin V and propidium iodide (PI) according to manufacturer instructions (Invitrogen, Thermo Fisher Scientific). Cells were analyzed via flow cytometry (CytoFLEX, Beckman Coulter), and unstained samples were used to gate neutrophils based on size (forward scatter) and granularity (side scatter). Quadrants were defined using cells singly stained with annexin V or PI. Populations of live (annexin V^−^/PI^−^), apoptotic (annexin V^+^/PI^−^), and dead (annexin V^+^/PI^+^) neutrophil singlets were quantified based upon at least 10,000 events. Post-acquisition data analysis was performed using commercial software (FlowJo v. 10.7.1). Raw percentages of neutrophil viability, apoptosis, and death are available in Supplementary Figures S2, S3. For data analysis, the percentages of live, apoptotic, or dead neutrophils were expressed relative to media control, unprimed neutrophils.

### Statistical Analyses

For functional assays, one-tailed paired *t*-tests were performed to compare VC-treated neutrophils under stimulated vs. unstimulated conditions. Withaferin A treatment groups and the media control group were compared to the VC group within each stimulation condition using one-way repeated measures ANOVA with *post hoc* Holm–Šidák multiple comparisons. The WFA concentration at which 50% inhibition of each function was achieved relative to the VC (IC_50_) was determined using commercial software based upon four parameter nonlinear fit.

For apoptosis/viability data, apoptosis or viability of media control neutrophils under GM-CSF-primed conditions was compared to that of media control, unprimed neutrophils using one-tailed paired *t*-tests. Results from WFA-, VC-, or staurosporine positive control-treated neutrophils were compared to findings from media control neutrophils within each priming condition using one-way repeated measures ANOVA with *post hoc* Holm–Šidák multiple comparisons. All analyses described in this section were performed using GraphPad Prism v. 9.3.0. Significance was set at *p* < 0.05 for all analyses.

## Results

### Withaferin A Inhibits Adhesion of Activated Equine Neutrophils

**Figure 1:**
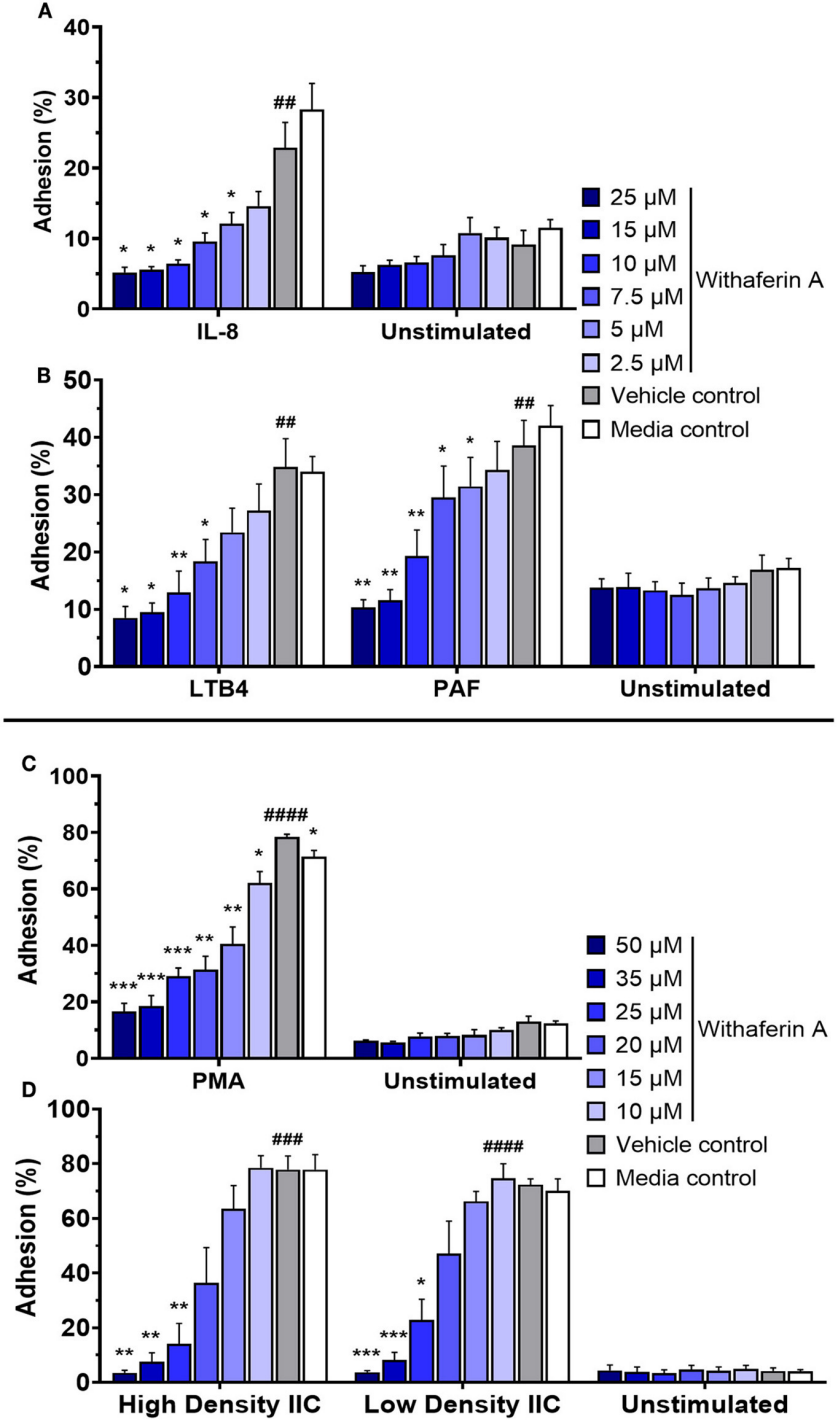
Withaferin A inhibits neutrophil adhesion to substrate in response to PMA, insoluble immune complexes (IIC), IL-8, LTB4, and PAF. Mean (SEM) percent adhesion of treated equine neutrophils to 5% FBS-coated plastic with IL-8 [**(A)** *n* = 4 horses], LTB4 [**(B)** *n* = 5 horses], PAF [**(B)** *n* = 5 horses], or PMA [**(C)** *n* = 5 horses] stimulation or to IIC immobilized to plastic [**(D)** *n* = 5 horses]. Fluorescent detection of calcein AM-loaded neutrophils after serial washes. One-tailed paired t-test of VC neutrophils; ^##^*p* < 0.01, ^###^*p* < 0.001, ^####^*p* < 0.0001 compared to unstimulated VC. One-way repeated measures ANOVA (Holm-Sidak multiple comparison testing); ^*^p < 0.05, ^**^p < 0.01, ^***^p < 0.001, as compared to VC within same stimulation condition.

Adhesion of circulating neutrophils to endothelial ligands is a critical initial event in neutrophilic inflammation. Using a modified protocol previously established by our laboratory, we evaluated the effect of WFA on neutrophil static adhesion induced by IL-8, LTB4, PAF, PMA, or IIC (74, 75). Each stimulus induced significant adhesion of media control neutrophils compared to unstimulated conditions (Figure 1). Treatment of neutrophils with WFA 10 min prior to stimulation caused a concentration-dependent inhibition of adhesion across all five stimuli relative to the neutrophils treated with VC (Figure 1). At the highest concentrations of WFA tested for each stimulus, percent adhesion of stimulated neutrophils approximated the percent adhesion of unstimulated, media control neutrophils. The adhesion IC_50_ values ranged from 6.8 to 21.3 μM (IL-8: 6.8 μM, LTB4: 7.5 μM, PAF: 9.1 μM, PMA: 12.4 μM, high density IIC: 18.5, low density IIC 21.3 μM). There was no significant effect of WFA on adhesion of unstimulated neutrophils. There was a small but statistically significant difference between percent adhesion of VC-treated neutrophils (78 ± 2.1%) compared to media control (71 ± 4.8%) with PMA stimulation. This was not observed with the other stimuli.

### Withaferin A Suppresses Neutrophil Migration Toward IL-8, LTB4, and PAF

**Figure 2:**
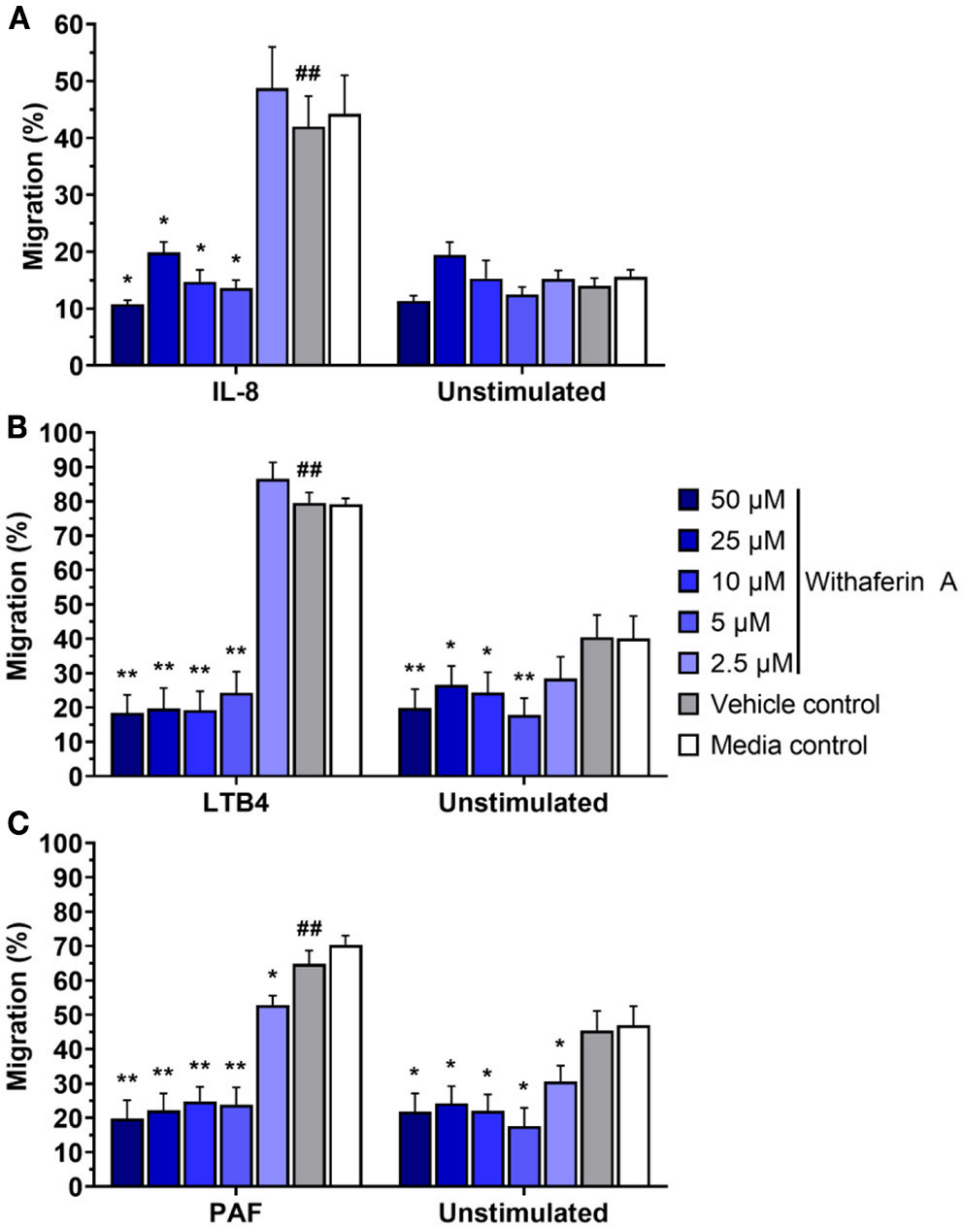
Withaferin A inhibits neutrophil migration toward IL-8, LTB4, and PAF. Mean (SEM) percent migration of treated equine neutrophils toward IL-8 [**(A)** *n* = 5 horses], LTB4 [**(B)** *n* = 4 horses], or PAF [**(C)** *n* = 4 horses]. Fluorescent detection of calcein AM-loaded neutrophils relative to 100% migration control wells. One-tailed paired t-test of VC neutrophils; ^##^*p* < 0.01 compared to unstimulated VC. One-way repeated measures ANOVA (Holm-Sidak multiple comparison testing); *p < 0.05, **p < 0.01, as compared to VC within same stimulation condition.

Next, we investigated the effect of WFA on neutrophil migration. The capacity of neutrophils to migrate toward chemoattractants was assessed using a commercial chemotaxis system and fluorescently-labeled neutrophils (74, 75). Withaferin A pretreatment of equine neutrophils significantly suppressed migration toward IL-8, LTB4, and PAF compared to pretreatment with VC (Figure 2). The magnitude of migration inhibition was marked, with concentrations of WFA as low as 5 μM suppressing percent migration of stimulated neutrophils below that of unstimulated, VC- or media-treated neutrophils. Migration IC_50_ values for IL-8, LTB4, and PAF stimulation were 2.5 μM, 2.9 μM, and 1.0 μM, respectively. Withaferin A also decreased neutrophil migration in the absence of stimulation relative to the VC treatment (Figures 2B,C). There were no differences between migration of VC or media control neutrophils under any stimulation condition.

### Withaferin A Inhibits Neutrophil Respiratory Burst in Response to GM-CSF/LPS, PMA, and Insoluble Immune Complexes

**Figure 3:**
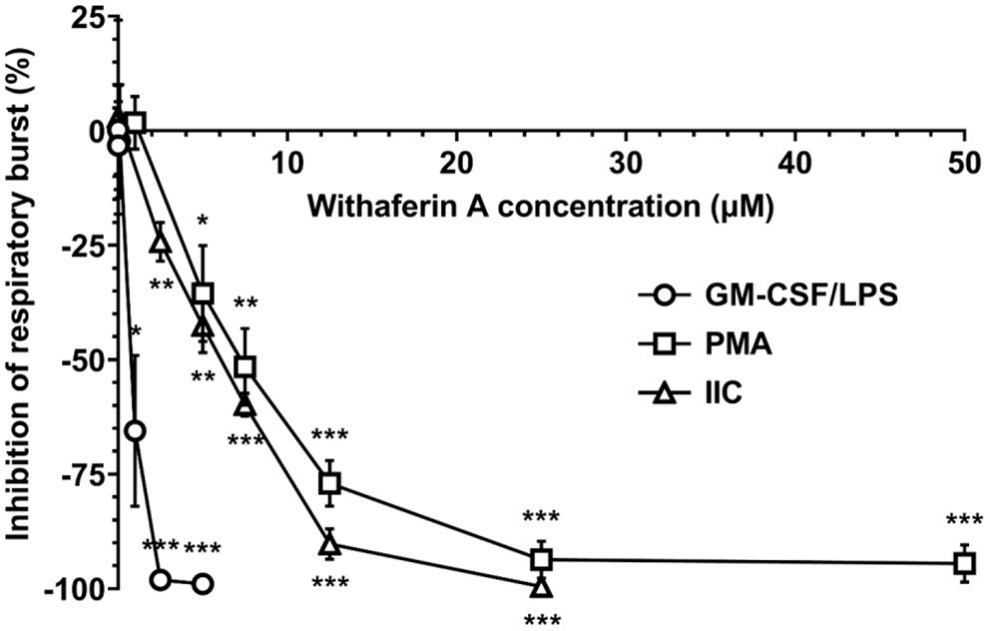
Withaferin A inhibits neutrophil respiratory burst stimulated by GM-CSF/LPS, PMA, and insoluble immune complexes (IIC). Mean (SEM) percent difference in mean luminescence relative to VC in WFA-treated equine neutrophils post-stimulation with GM-CSF/LPS (circles, 40 min, *n* = 6 horses), PMA (squares, 60 min, *n* = 5 horses), or IIC (diamonds, 30 min, *n* = 5 horses). Luminol-enhanced chemiluminescence detection of reactive oxygen species. One-way repeated measures ANOVA (Holm-Sidak multiple comparison testing); *p < 0.05, **p < 0.01, ***p < 0.001, as compared to 0% difference from VC.

After demonstrating that WFA interferes with key steps corresponding to neutrophil extravasation, we next sought to determine the effect of WFA on production of reactive oxygen species, a major contributor to neutrophil-mediated host cytotoxicity. We quantified the production of reactive oxygen species via luminol-enhanced chemiluminescence (74). Results were expressed as percent inhibition relative to VC-treated neutrophils under each stimulation condition. Figure 3 demonstrates a concentration-dependent inhibition of respiratory burst by WFA compared to the treatment VC for all stimulation conditions. Respiratory burst IC_50_ values for GM-CSF/LPS, PMA, and IIC stimulation were 0.9 μM, 6.5 μM, and 6.5 μM, respectively. Withaferin A also significantly reduced the luminescence AUC in PMA- and IIC-stimulated neutrophils compared to vehicle control (Supplementary Figures S4A,B). The decrease in luminescence AUC in WFA-treated, GM-CSF/LPS-stimulated neutrophils did not reach significance due to variability in vehicle control luminescence magnitude between independent experiments (Supplementary Figure S4C). Production of reactive oxygen species was low in unstimulated neutrophils and was not significantly affected by WFA (Supplementary Figures S1, S4). There was no significant difference between respiratory burst of VC and media control neutrophils under stimulated conditions (Supplementary Figure S4).

### Withaferin A Does Not Compromise Short-Term Neutrophil Viability but Abrogates Delayed Apoptosis in Primed Neutrophils

**Figure 4:**
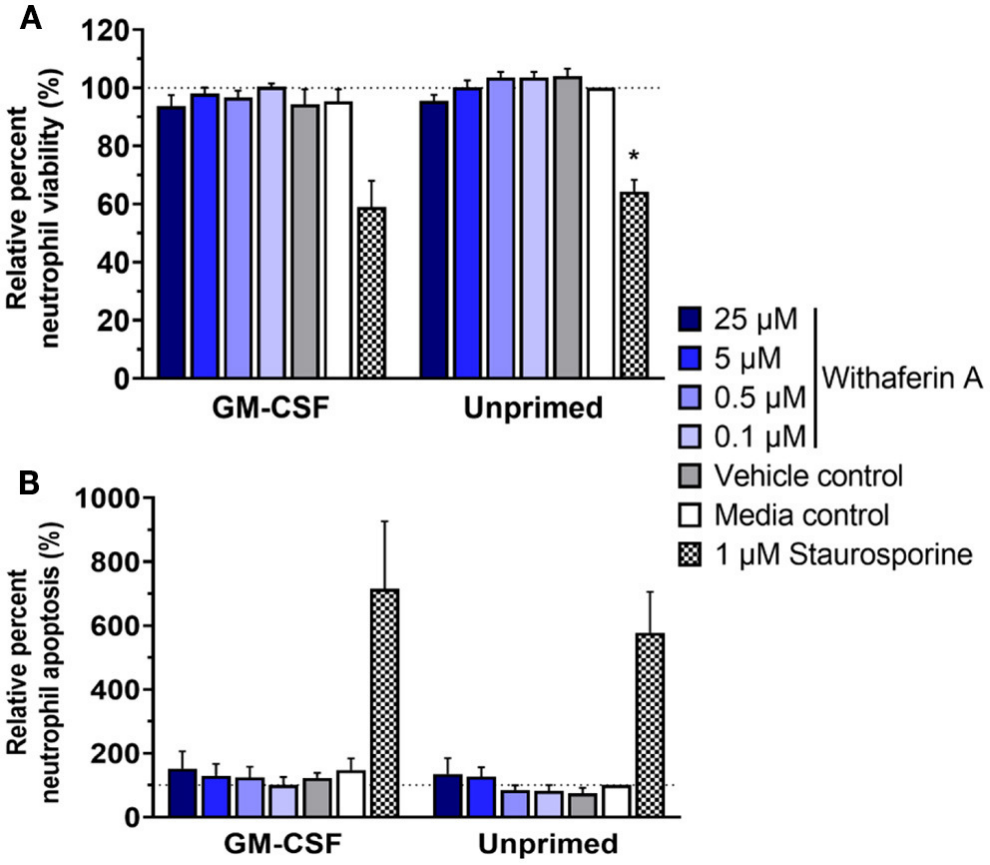
Withaferin A does not impair short-term neutrophil viability or cause rapid neutrophil apoptosis. Mean (SEM) percentage of live **(A)** and apoptotic **(B)** neutrophils relative to media control, unprimed neutrophils following 2-h treatment of equine neutrophils under GM-CSF-primed (*n* = 3 horses) or unprimed (*n* = 4 horses) conditions. Flow cytometry quantification of annexin V binding and propidium iodide staining. One-way repeated measures ANOVA (Holm-Sidak multiple comparison testing); *p < 0.05 compared to media control within same priming condition.

Next, we investigated the possibility of cell death or apoptosis as an explanation for WFA inhibition of neutrophil functions. We incubated neutrophils with trypan blue at the end of respiratory burst assays: following 30 min of 50 μM WFA pretreatment and an additional 90 min of stimulation. Viability of treated neutrophils, as assessed by ability to exclude trypan blue dye, was consistently ≥98% (data not shown). This finding suggests that WFA treatment does not suppress neutrophil functions by causing neutrophil cell death. However, we recognize that compromise of plasma membrane is a relatively late event in the process of cell death, so inability to exclude trypan blue has limited sensitivity for evaluating cell viability. Trypan blue staining also does not identify cells in early apoptosis. Therefore, we used flow cytometry to assess neutrophil viability using a more stringent definition of live neutrophils (annexin V^−^/propidium iodide^−^) and to quantify apoptotic neutrophils (annexin V^+^/propidium iodide^−^). After 2 h incubation in the presence or absence of GM-CSF priming, the percentage of live neutrophils was not different in WFA-treated cells (0.1–25 μM WFA) compared to media control-treated cells (Figure 4A). Similarly, 2-h treatment with WFA did not significantly increase apoptosis nor cell death relative to media control in GM-CSF-primed or unprimed neutrophils (Figure 4B, Supplementary Figure S5).

**Figure 5:**
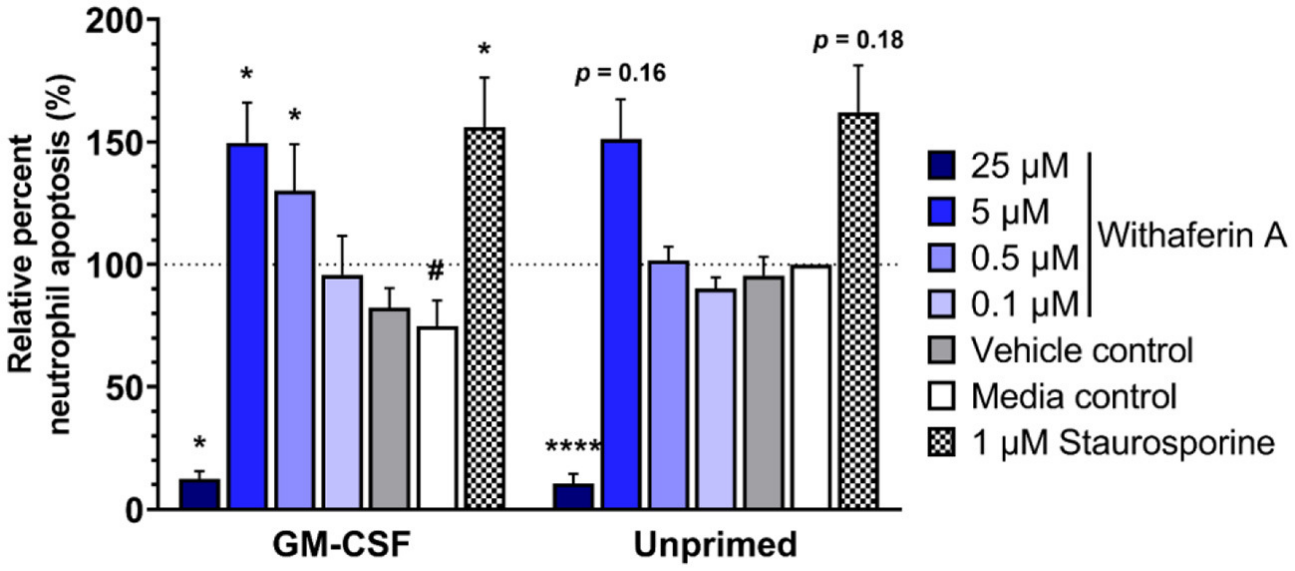
Withaferin A promotes apoptosis of primed neutrophils by 24 h. Mean (SEM) percentage of apoptotic neutrophils relative to media control, unprimed neutrophils following 24-h treatment of equine neutrophils under GM-CSF-primed or unprimed conditions (*n* = 6 horses). Paired *t*-test of media control neutrophils; ^#^*p* < 0.05 compared to unprimed media control. One-way repeated measures ANOVA (Holm-Sidak multiple comparison testing); *p < 0.05, ****p < 0.0001 compared to media control within same priming condition.

We next evaluated the effect of WFA on 24-h neutrophil apoptosis under primed or unprimed conditions. Exposure of media control neutrophils to GM-CSF for 24 h decreased neutrophil apoptosis by 25% relative to unprimed conditions, confirming the pro-survival effects of GM-CSF in our model (Figure 5, Supplementary Figure S6A). As expected, staurosporine (positive control) significantly increased the percentage of apoptotic cells compared to media treatment. Similarly, and consistent with our hypothesis, neutrophils exposed to GM-CSF and treated with 5 μM or 0.5 μM WFA had significantly higher rates of apoptosis compared to media control, suggesting a pro-apoptotic effect of WFA under these conditions (Figure 5). Interestingly, the effect of WFA or staurosporine on increasing neutrophil apoptosis did not reach significance in neutrophils that were not exposed to GM-CSF. Regardless of GM-CSF exposure, 24-h treatment with 25 μM WFA was associated with a significant reduction in apoptosis, reflective of an increased percentage of dead cells that were annexin V^+^/propidium iodide^+^ (Supplementary Figure S6B). The VC had no effect on neutrophil apoptosis under primed or unprimed conditions.

## Discussion

Neutrophils are central to the pathophysiology of several common equine diseases, though current therapeutics that target neutrophilic inflammation are limited. Withaferin A demonstrates promise as a therapeutic agent for inflammatory diseases and mitigates pathology in animal models of neutrophil-mediated conditions. However, the effects of WFA on neutrophils have not been well described. For this study, our objectives were to determine the effects of WFA on equine neutrophil functions and apoptosis. Based on previously reported anti-inflammatory effects of WFA in neutrophil-mediated diseases, we hypothesized that WFA would inhibit adhesion, migration, and respiratory burst of equine neutrophils and would promote apoptosis of primed equine neutrophils.

For this project, we used multiple stimuli that utilize different signaling pathways to show that WFA significantly attenuates neutrophil adhesion, migration, and respiratory burst. Each of these neutrophil functions contributes to tissue damage, underscoring their relevance as potential therapeutic anti-inflammatory targets. These novel findings expand upon previous publications containing limited descriptions of WFA effects on leukocyte functions. Specifically, Che et al. reported that WFA inhibited *Escherichia coli*-induced neutrophil migration (58), while Sabina et al. showed decreased *in vitro* degranulation of WFA-treated human neutrophils (45). Lee et al. showed that WFA treatment of cultured primary human endothelium suppressed adhesion and migration of monocytes (THP-1), which the authors attributed to reduced expression of endothelial cell adhesion molecules (77). Finally, while our report is the first to examine the effect of WFA on neutrophil adhesion and respiratory burst, a withanolide compound with similar structure to WFA was shown to inhibit neutrophil respiratory burst stimulated by combined N-formyl-methionyl-leucyl-phenyl-alanine/cytochalasin B (78).

Once isolated, the majority of neutrophils undergo constitutive apoptosis *in vitro* within 24 h (23, 79), mirroring the short *in vivo* lifespan of blood neutrophils in healthy subjects (21). However, exposure to GM-CSF, which occurs during many inflammatory conditions *in vivo*, reduces the rate of apoptosis and prolongs neutrophil survival (23). Therefore, 24-hour incubation of neutrophils with GM-CSF represents a physiologically relevant *in vitro* model for delayed neutrophil apoptosis under inflammatory conditions. The additional 2-hour time point for apoptosis experiments served two purposes. First, it allowed us to better understand the timeline of WFA's effects on neutrophil apoptosis. Second, it helped to validate the trypan blue viability data from our functional assays.

Consistent with previous reports, in this study, exposure to GM-CSF for 24 h reduced neutrophil apoptosis and enhanced neutrophil survival (23, 80, 81). Withaferin A treatment significantly increased apoptosis in primed neutrophils, mitigating the pro-survival effects of GM-CSF. The promotion of neutrophil apoptosis by WFA stands in contrasts to the anti-apoptotic effect of glucocorticoids on neutrophils (82, 83), which is often cited as a downside to glucocorticoid therapy (84, 85). Interestingly, in unprimed neutrophils, WFA did not have a significant pro-apoptotic effect. This finding suggests that WFA promotion of neutrophil apoptosis was mediated through inhibitory effects on anti-apoptotic/pro-survival signaling that occurs in primed neutrophils, rather than direct action on apoptotic machinery. Based on interpretation of our data, we further hypothesize that WFA interferes with pro-inflammatory signaling pathways that antagonize constitutive neutrophil apoptosis, facilitating a pro-apoptotic balance. This potential mechanism offers a plausible explanation of the differential effects of WFA on apoptosis of neutrophils and tumor cells (pro-apoptotic) (60, 61, 65, 66) vs. most non-neoplastic cells (anti-apoptotic) (70–73).

Withaferin A had no significant effect on neutrophil apoptosis at 2 h, regardless of GM-CSF exposure. This finding suggests that the overall pro-apoptotic effects of WFA in neutrophils are not rapid in onset. This would fit with a proposed mechanism of interference with anti-apoptotic signaling as opposed to direct activation of caspases. These data also exclude apoptosis as an explanation for our demonstrated suppression of neutrophil function. In accordance with the lack of effect of WFA on neutrophil apoptosis after 2 h, we also found that viability of WFA-treated neutrophils was not significantly different than media control at this timepoint. Taken together with neutrophil function data, we have established that WFA inhibition of neutrophil adhesion, migration, and respiratory burst is not mediated through a decrease in neutrophil viability.

The cellular mechanisms underlying WFA's effects on neutrophil inflammatory functions and apoptosis are currently unknown. Our 2-h flow cytometry viability data indicate that WFA suppression of multiple neutrophil functions is not attributable to cell death or apoptosis. The marked suppression of all neutrophil functions evaluated in this study provides further insight into potential mechanisms of action of WFA in neutrophils. Universal inhibition of neutrophil adhesion, migration, and respiratory burst suggests that WFA is preventing one or more events that are central to overall neutrophil activation, such as activation of β2 integrins on the neutrophil surface, reorganization of actin cytoskeletal structure, and/or regulation of intracellular calcium (12). Additionally, WFA's ability to suppress neutrophil responses to multiple stimuli also informs possible mechanisms of action. The diverse stimuli utilized in our functional assays signal through multiple cell signaling pathways. Therefore, the ability of WFA to inhibit responses to a variety of stimuli suggests that WFA is interfering with cell signaling that is essential for all of these methods of neutrophil stimulation.

There are several reported mechanisms of action for WFA effects in other cell types. Described mechanisms that are potentially relevant to our findings include inhibition of phosphorylation of major kinases and interference with nuclear factor kappa B (NF-κB) signaling (86–91). One or more mitogen-activated protein kinases (MAPKs), including extracellular signal-regulated kinase (ERK1/2), p38 MAPK, and/or c-JUN N-terminal kinase (JNK), are phosphorylated following priming of neutrophils with GM-CSF (92) or stimulation with IL-8 (93), LTB4 (94), PAF (95), PMA (96), LPS (97), and IIC (98). Many of these stimuli also trigger phosphorylation of Akt in neutrophils (98–102). Although the relative involvement of the different MAPKs and Akt in neutrophil functions are stimulus-dependent, all four are known to play a role in neutrophil adhesion, migration, and/or respiratory burst, as evidenced by decreases in neutrophil functionality after treatment with specific inhibitors of ERK1/2, p38 MAPK, JNK, and/or Akt (94, 103–105). Our laboratory and other groups have confirmed roles of these signaling pathways in equine neutrophil functions (94, 106, 107). Delayed neutrophil apoptosis during inflammation is mediated through ERK1/2 (81, 108), Akt (81, 108–110), and NF-κB (110) signaling pathways. Withaferin A interference with these signaling molecules could explain the increased apoptosis we observed in GM-CSF-primed neutrophils. While there is clear evidence from other cell types that WFA interferes with signaling molecules that regulate neutrophil function and lifespan, it is important to note that conflicting data (i.e., WFA enhancement of kinase activation) has also been reported in both neoplastic and nonneoplastic cells (49, 70, 111). These discrepancies underscore the need for additional work to determine the effects of WFA on these molecular targets in neutrophils and their relation to our findings presented here.

We recognize several limitations of this study. First, neutrophils used in the functional assays were pretreated with WFA for 10 min (adhesion experiments) or 30 min (migration and respiratory burst experiments) prior to stimulation. This timeline does not replicate clinical conditions in which WFA may be a valuable therapeutic, since treatment administration prior to the onset of inflammation is generally not feasible for acute diseases. However, it is important to note that inflammatory events, particularly those involving dysregulated neutrophils responses, are cyclical, with initial neutrophilic inflammation contributing to tissue damage that often recruits additional neutrophils. In these situations, anti-inflammatories such as WFA might be useful for “interrupting” a dysregulated cycle of inflammation. Additionally, our 24-h apoptosis findings suggest that WFA may actively promote resolution of existing inflammation. In other scenarios, particularly for diseases that are chronic with episodes of exacerbation, WFA could be used in a chemopreventive capacity to reduce inflammatory flares. Second, our experiments were conducted with isolated neutrophils containing a low percentage of other leukocyte populations. While this reductionist approach is essential for examining direct effects of WFA on neutrophils to better understand mechanisms, it ignores the effects of WFA on other cell types (e.g., leukocytes, epithelium, endothelium, etc.) and the resulting complex interactions that may influence neutrophils indirectly. We plan to incorporate other relevant cell types into future experiments in preparation for subsequent *in vivo* WFA studies. Third, our population of dead neutrophils was defined by cells that were double positive for annexin V and propidium iodide staining and includes both late apoptotic and necrotic cells. We attempted to differentiate the two processes using a pan-caspase inhibitor, z-VAD-FMK, but none of the three concentrations of z-VAD-FMK previously reported to inhibit neutrophil apoptosis (20 μM, 50 μM, 100 μM z-VAD-FMK) (80, 102, 112) had an effect on the percentage of annexin V^+^/propidium iodide^−^ neutrophils in our experiments (data not shown). Therefore, we were unable to determine whether the higher percentage of dead neutrophils following 25 μM WFA treatment for 24 h was reflective of an earlier onset and/or more rapid progression of apoptosis leading to secondary loss of membrane integrity or whether the 25 μM WFA caused necrosis rather than apoptosis. Since necrosis is an inherently pro-inflammatory process, understanding the potential of this higher concentration of WFA to cause necrosis of neutrophils is relevant to its future clinical use. Finally, we base our assertion that WFA does not compromise short-term neutrophil viability on flow cytometry data from neutrophils simultaneously treated with WFA and primed with GM-CSF or left unprimed for 2 h. The 2-h treatment time exceeds the length of exposure to WFA during our functional assays (total WFA exposure ranged from 40 to 90 min). However, we acknowledge the possibility that stimuli used in the functional assays may affect impact of WFA on neutrophil viability. We repeated apoptosis/cell death flow cytometry experiments using similar conditions as adhesion and respiratory burst assays, including a 30-min WFA pretreatment period followed by stimulation with PMA or GM-CSF/LPS or no stimulation (30–60 min). There was no evidence that WFA reduced neutrophil viability under conditions used in functional assays (data not shown).

Here we report exciting *in vitro* results that provide proof of principle evidence for WFA as a novel anti-inflammatory strategy for neutrophil-mediated diseases. We have shown that WFA inhibits neutrophil adhesion, migration, and respiratory burst across a wide range of stimuli, including those that are relevant to inflammatory diseases, through a mechanism that does not impair short-term neutrophil viability. We also demonstrate that WFA promotes timely apoptosis of GM-CSF-primed neutrophils, a rare example of pro-apoptotic effects of WFA in non-neoplastic cells. The combination of these effects may have clinical benefit in reducing inflammation and hastening resolution of inflammation. Further studies on WFA are needed to better understand the molecular mechanisms underlying these effects, confirm the favorable safety profile suggested by laboratory animal and human data (113, 114), and evaluate feasibility and optimal delivery in veterinary species and humans.

## Data Availability Statement

The raw data supporting the conclusions of this article will be made available by the authors, without undue reservation.

## Ethics Statement

The animal study was reviewed and approved by North Carolina State University Institutional Animal Care and Use Committee (IACUC protocol #19-779).

## Author Contributions

RB: conceptualization, study design, funding acquisition, study execution, data analysis and interpretation, original draft preparation, manuscript review and editing, and final approval. MS: supervision, data interpretation, manuscript review and editing, and final approval. SJ: conceptualization, study design, funding acquisition, supervision, data interpretation, manuscript review and editing, and final approval. All authors contributed to the article and approved the submitted version.

## Funding

Stipend support for RB and partial funding for study supplies were provided by the NIH T32 Ruth Kirschstein Institutional National Research Service Award (NRSA) Training Grant (NIH T32 OD011130). Other sources of funding for experiments included the North Carolina State University College of Veterinary Medicine Competitive Research Grants Program and the North Carolina State University Comparative Medicine Institute Associate Member Flash Grant Program.

## Conflict of Interest

The authors declare that the research was conducted in the absence of any commercial or financial relationships that could be construed as a potential conflict of interest.

## Publisher's Note

All claims expressed in this article are solely those of the authors and do not necessarily represent those of their affiliated organizations, or those of the publisher, the editors and the reviewers. Any product that may be evaluated in this article, or claim that may be made by its manufacturer, is not guaranteed or endorsed by the publisher.

## Supplementary Material

Supplementary Figure S1 Kinetics of neutrophil respiratory burst are stimulus dependent. Mean (SEM) percent luminescence of media control neutrophils stimulated with GM-CSF/LPS [**(A)** circles, *n* = 6 horses], PMA [**(B)** squares, *n* = 5 horses], or IIC [**(C)** diamonds, *n* = 5 horses] or appropriate stimulus controls **(A–C)** black diamonds), relative to maximum luminescence of stimulated neutrophils during assay time course. Luminol-enhanced chemiluminescence detection of reactive oxygen species. Vertical dashed line indicates time point selected to compare effect of treatments under each stimulation condition. One-tailed paired *t*-test at selected time point; ^####^*p* < 0.0001 compared to unstimulated control.

Supplementary Figure S2 Raw percentages of neutrophil viability, apoptosis, death after 2-h incubation. Mean (SEM) percentages of live **(A)**, apoptotic **(B)** and dead **(C)** neutrophils following 2-h treatment of equine neutrophils under GM-CSF-primed (*n* = 3 horses) or unprimed (*n* = 4 horses) conditions. Flow cytometry quantification of annexin V binding and propidium iodide staining. No statistical analyses performed on raw data.

Supplementary Figure S3 Raw percentages of neutrophil viability, apoptosis, death after 24-h incubation. Mean (SEM) percentages of live **(A)**, apoptotic **(B)** and dead **(C)** neutrophils following 24-h treatment of equine neutrophils under GM-CSF-primed or unprimed (*n* = 6 horses) conditions. Flow cytometry quantification of annexin V binding and propidium iodide staining. No statistical analyses performed on raw data.

Supplementary Figure S4 Withaferin A decreases overall respiratory burst in neutrophils stimulated with PMA or insoluble immune complexes (IIC). Mean (SEM) luminescence area under the curve (AUC) in neutrophils pretreated with WFA, VC, or media control under PMA [**(A)** *n* = 5 horses], IIC [**(B)** *n* = 5 horses], or GM-CSF/LPS [**(C)** *n* = 6 horses] stimulation. Luminol-enhanced chemiluminescence detection of reactive oxygen species for 90 minutes post-stimulation. One-tailed paired *t*-test of VC neutrophils; ^###^*p* < 0.001 compared to unstimulated VC. One-way repeated measures ANOVA (Holm-Sidak multiple comparison testing); ^*^*p* < 0.05, ^**^*p* < 0.01, as compared to VC within same stimulation condition.

Supplementary Figure S5 Withaferin A does not cause rapid neutrophil cell death. Mean (SEM) percentage of dead neutrophils relative to media control, unprimed neutrophils following 2-h treatment of equine neutrophils under GM-CSF-primed (*n* = 3 horses) or unprimed (*n* = 4 horses) conditions. Flow cytometry quantification of annexin V binding and propidium iodide staining. Paired *t*-test of media control neutrophils; no significant difference between GM-CSF-primed and unprimed media control neutrophils. One-way repeated measures ANOVA (Holm-Sidak multiple comparison testing); no significant difference between treatments and media control within same priming condition.

Supplementary Figure S6 Higher concentrations of Withaferin A decrease neutrophil viability and increase neutrophil death by 24 h. Mean (SEM) percentage of live **(A)** and dead **(B)** neutrophils relative to media control, unprimed neutrophils following 24-h treatment of equine neutrophils under GM-CSF-primed or unprimed (*n* = 6 horses) conditions. Flow cytometry quantification of annexin V binding and propidium iodide staining. Paired *t*-test of media control neutrophils; ^#^*p* < 0.05, ^####^*p* < 0.0001 compared to unprimed media control. One-way repeated measures ANOVA (Holm-Sidak multiple comparison testing); ^*^*p* < 0.05, ^**^*p* < 0.01, ^***^*p* < 0.001 compared to media control within same priming condition.
