# Supplementary figures and images for: Withaferin A Inhibits Neutrophil Adhesion, Migration, and Respiratory Burst and Promotes Timely Neutrophil Apoptosis
Source: Front Vet Sci. 2022 Jun 17;9:900453. doi: 10.3389/fvets.2022.900453 (PMC9247543; doi:10.3389/fvets.2022.900453)

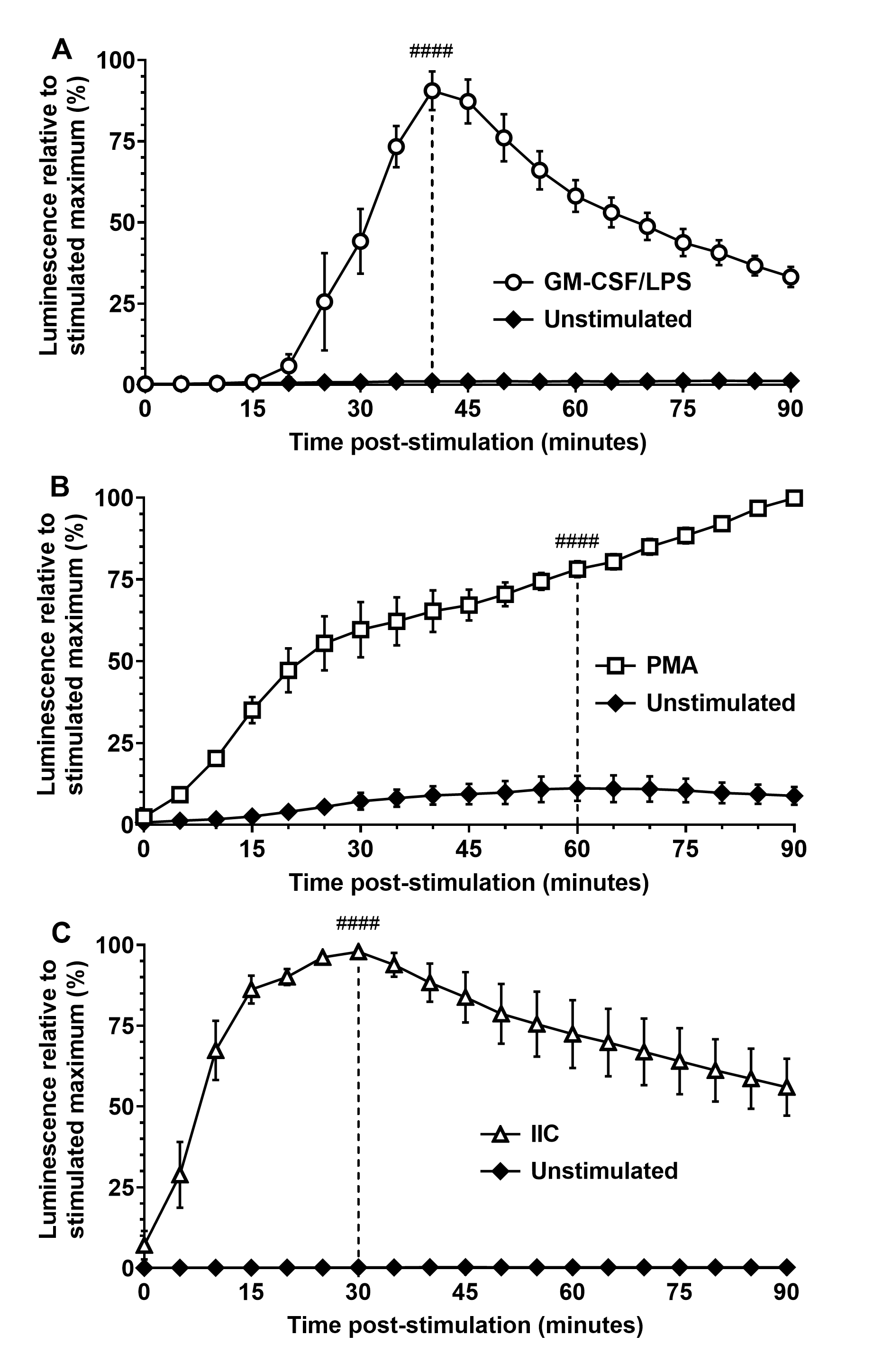

Supplement: Supplementary Figure S1 — Kinetics of neutrophil respiratory burst are stimulus dependent. Mean (SEM) percent luminescence of media control neutrophils stimulated with GM-CSF/LPS [(A) circles, n = 6 horses], PMA [(B) squares, n = 5 horses], or IIC [(C) diamonds, n = 5 horses] or appropriate stimulus controls (A–C) black diamonds), relative to maximum luminescence of stimulated neutrophils during assay time course. Luminol-enhanced chemiluminescence detection of reactive oxygen species. Vertical dashed line indicates time point selected to compare effect of treatments under each stimulation condition. One-tailed paired t-test at selected time point; ####p < 0.0001 compared to unstimulated control. [file Image_1.TIF]

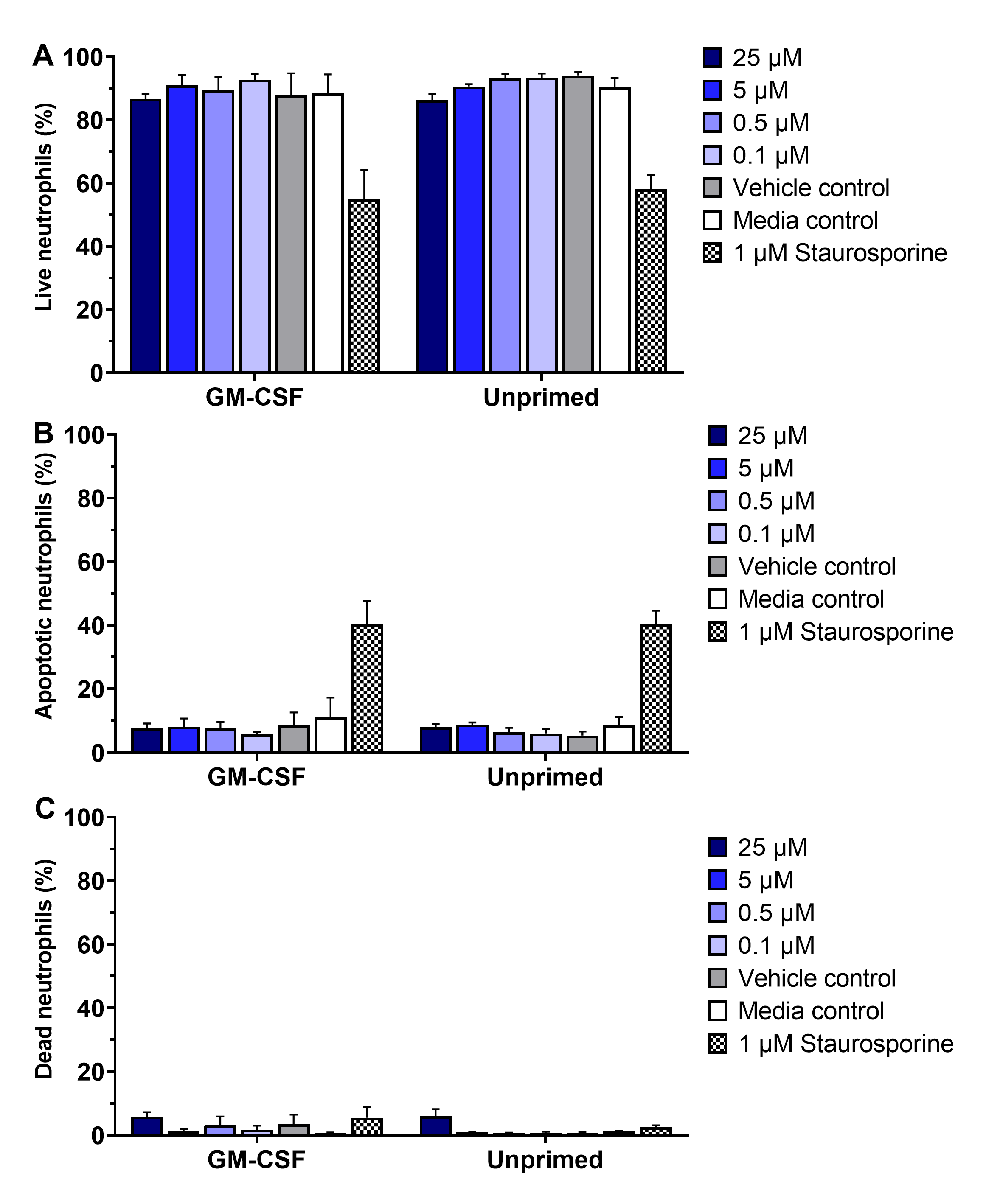

Supplement: Supplementary Figure S2 — Raw percentages of neutrophil viability, apoptosis, death after 2-h incubation. Mean (SEM) percentages of live (A), apoptotic (B) and dead (C) neutrophils following 2-h treatment of equine neutrophils under GM-CSF-primed (n = 3 horses) or unprimed (n = 4 horses) conditions. Flow cytometry quantification of annexin V binding and propidium iodide staining. No statistical analyses performed on raw data. [file Image_2.TIF]

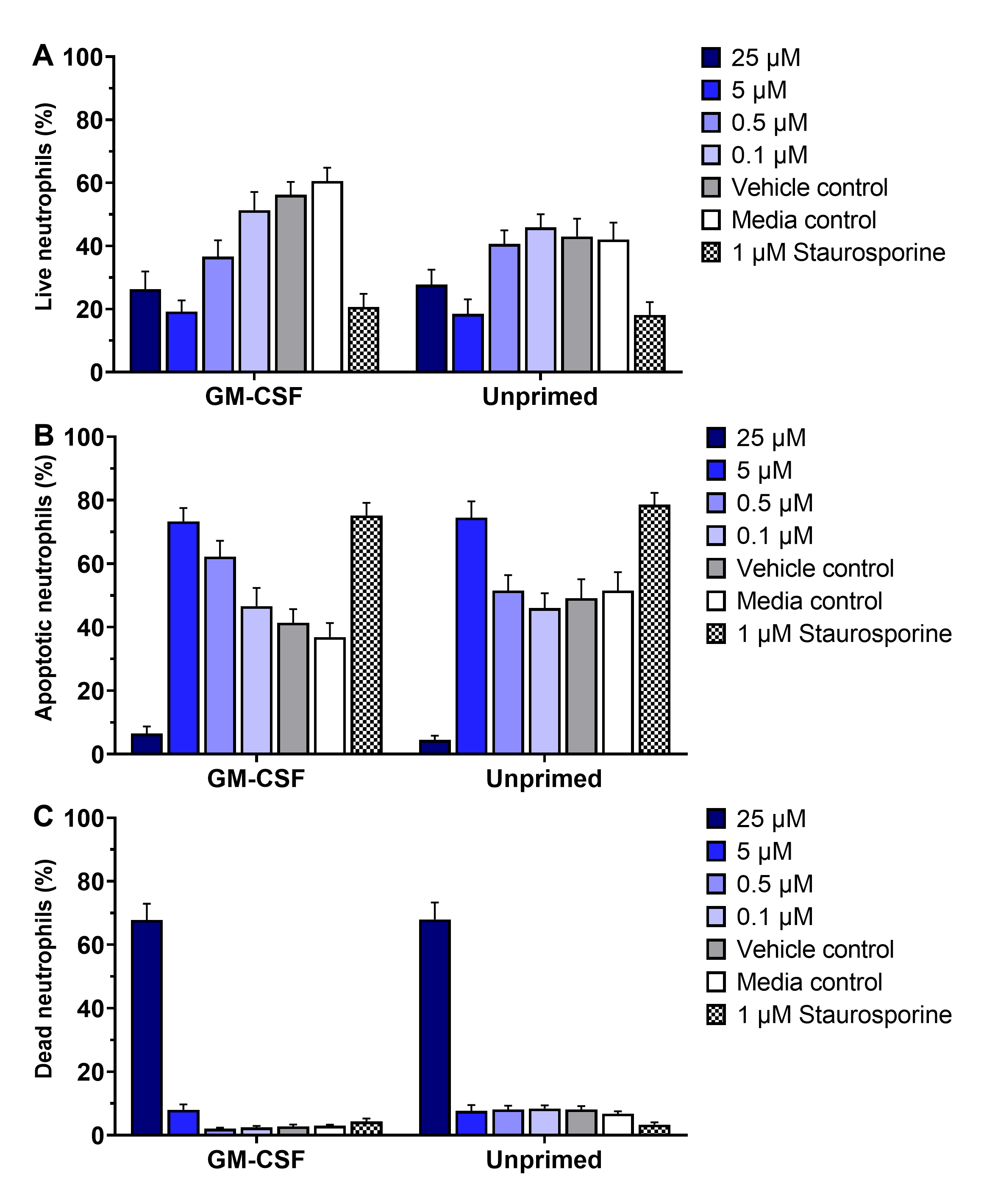

Supplement: Supplementary Figure S3 — Raw percentages of neutrophil viability, apoptosis, death after 24-h incubation. Mean (SEM) percentages of live (A), apoptotic (B) and dead (C) neutrophils following 24-h treatment of equine neutrophils under GM-CSF-primed or unprimed (n = 6 horses) conditions. Flow cytometry quantification of annexin V binding and propidium iodide staining. No statistical analyses performed on raw data. [file Image_3.TIF]

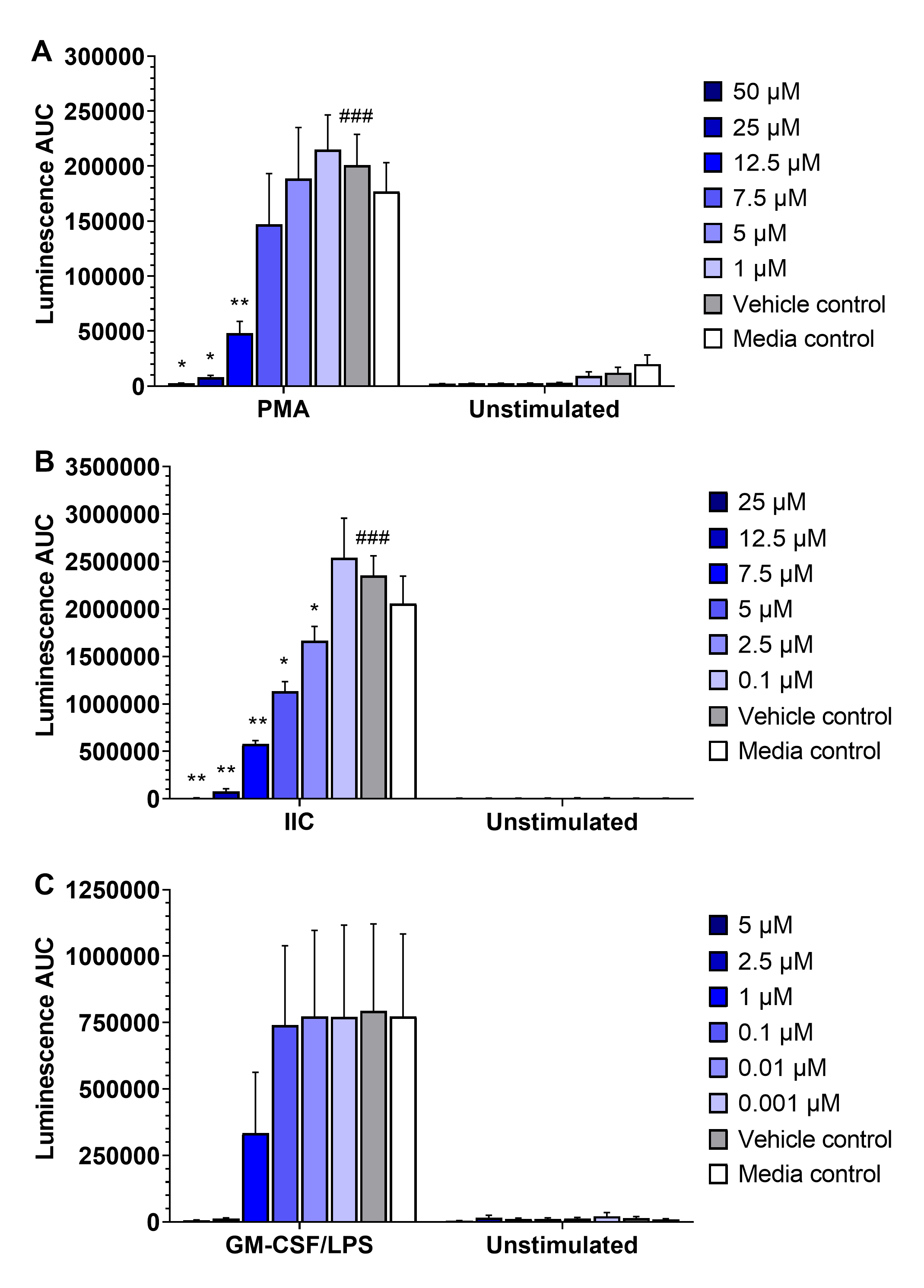

Supplement: Supplementary Figure S4 — Withaferin A decreases overall respiratory burst in neutrophils stimulated with PMA or insoluble immune complexes (IIC). Mean (SEM) luminescence area under the curve (AUC) in neutrophils pretreated with WFA, VC, or media control under PMA [(A) n = 5 horses], IIC [(B) n = 5 horses], or GM-CSF/LPS [(C) n = 6 horses] stimulation. Luminol-enhanced chemiluminescence detection of reactive oxygen species for 90 minutes post-stimulation. One-tailed paired t-test of VC neutrophils; ###p < 0.001 compared to unstimulated VC. One-way repeated measures ANOVA (Holm-Sidak multiple comparison testing); *p < 0.05, **p < 0.01, as compared to VC within same stimulation condition. [file Image_4.TIF]

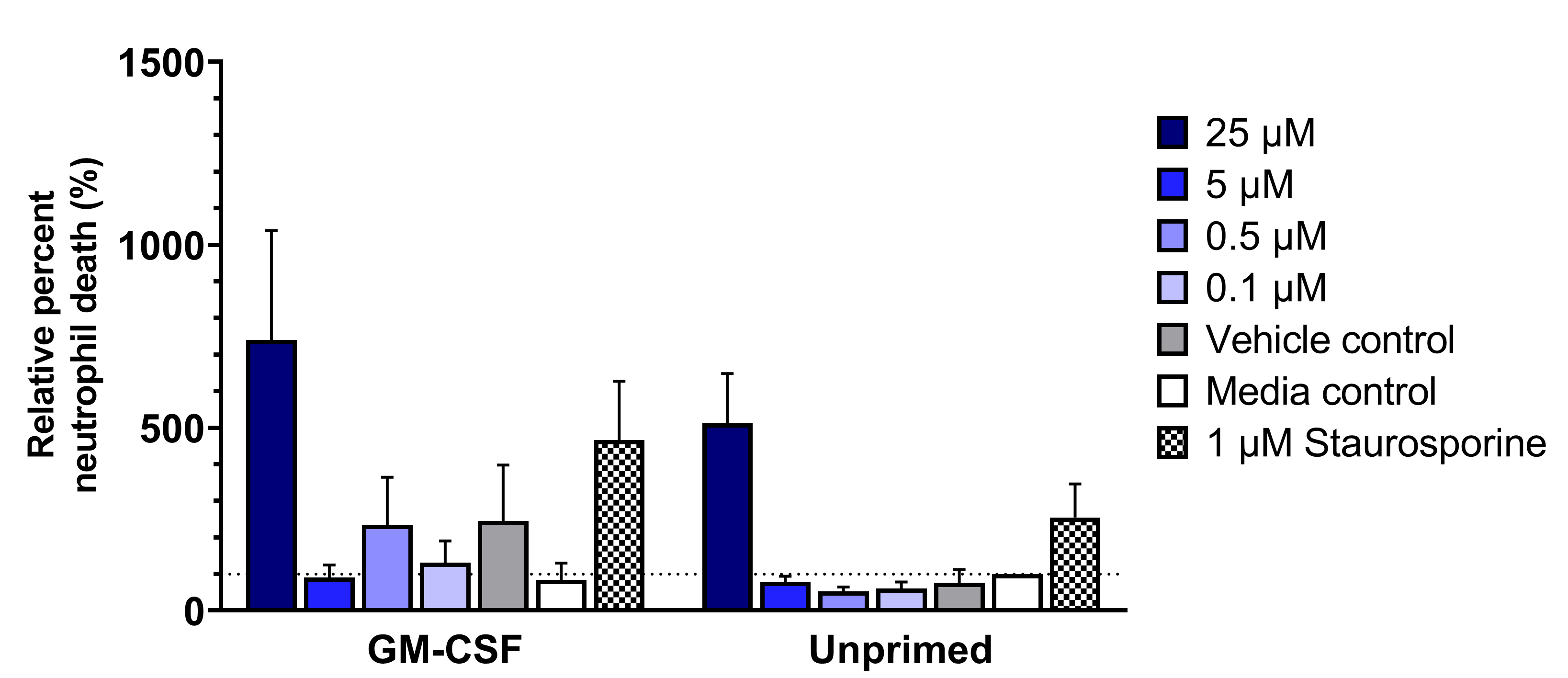

Supplement: Supplementary Figure S5 — Withaferin A does not cause rapid neutrophil cell death. Mean (SEM) percentage of dead neutrophils relative to media control, unprimed neutrophils following 2-h treatment of equine neutrophils under GM-CSF-primed (n = 3 horses) or unprimed (n = 4 horses) conditions. Flow cytometry quantification of annexin V binding and propidium iodide staining. Paired t-test of media control neutrophils; no significant difference between GM-CSF-primed and unprimed media control neutrophils. One-way repeated measures ANOVA (Holm-Sidak multiple comparison testing); no significant difference between treatments and media control within same priming condition. [file Image_5.TIF]

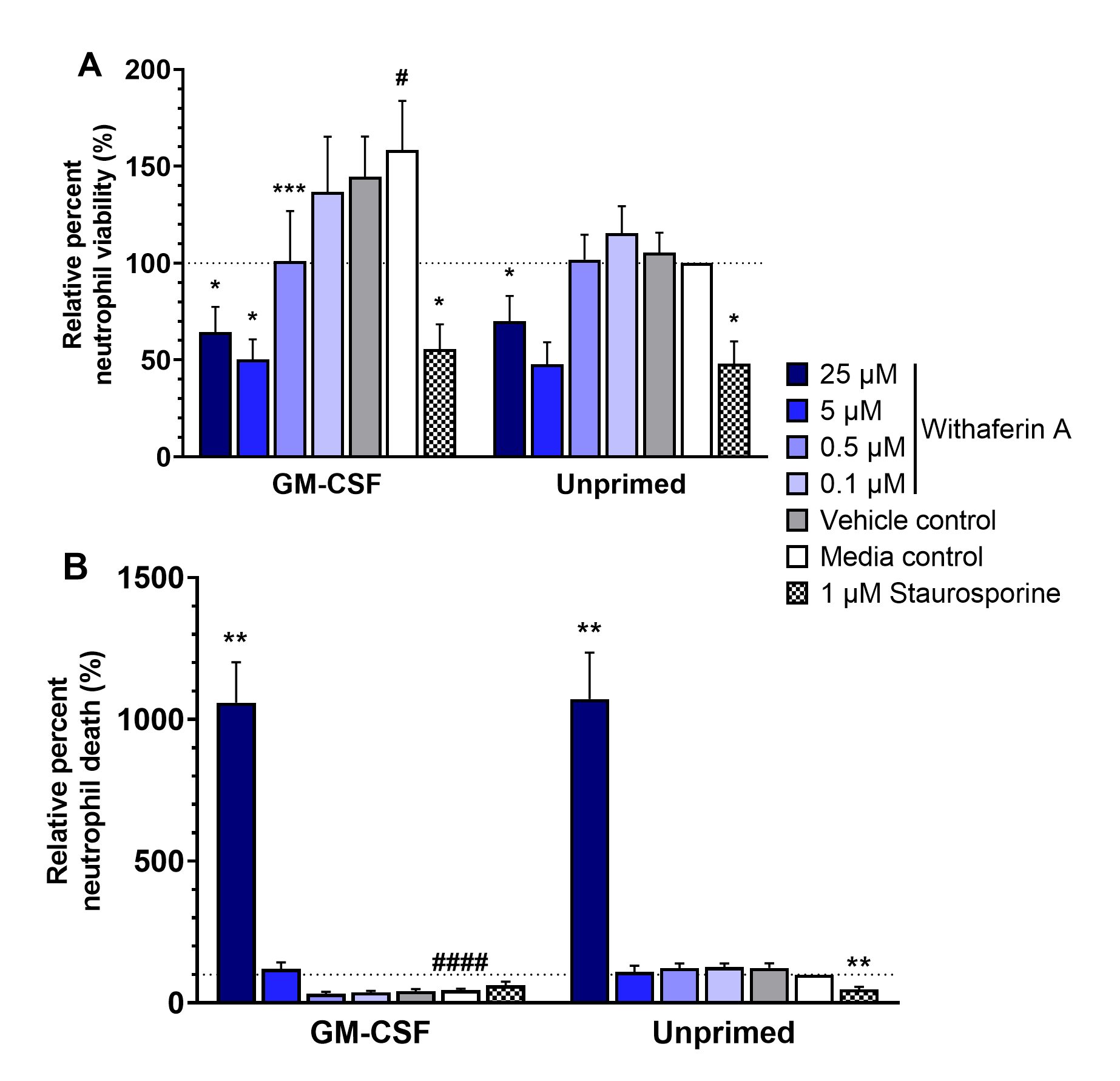

Supplement: Supplementary Figure S6 — Higher concentrations of Withaferin A decrease neutrophil viability and increase neutrophil death by 24 h. Mean (SEM) percentage of live (A) and dead (B) neutrophils relative to media control, unprimed neutrophils following 24-h treatment of equine neutrophils under GM-CSF-primed or unprimed (n = 6 horses) conditions. Flow cytometry quantification of annexin V binding and propidium iodide staining. Paired t-test of media control neutrophils; #p < 0.05, ####p < 0.0001 compared to unprimed media control. One-way repeated measures ANOVA (Holm-Sidak multiple comparison testing); *p < 0.05, **p < 0.01, ***p < 0.001 compared to media control within same priming condition. [file Image_6.TIF]
